# Early cytological diagnosis of extranodal stage I, primary thyroid Non-Hodgkin lymphoma in elderly patients. Report of two cases and review of the literature

**DOI:** 10.1186/1471-2482-13-S2-S49

**Published:** 2013-10-08

**Authors:** Elena Vigliar, Alessia Caleo, Mario Vitale, Vincenzo Di Crescenzo, Alfredo Garzi, Pio Zeppa

**Affiliations:** 1 Department of Medicine and Surgery, University of Salerno, Italy; 2 Department of Biomorphological and Functional Sciences, Faculty of Medicine and Surgery, University of Naples "Federico II", Italy

## Abstract

**Background:**

Primary thyroid lymphomas (PTLs) account for 5% of thyroid malignant tumors and often develop in patients with Hashimoto Thyroiditis (HT). Fine-needle cytology (FNC) is widely used in the diagnosis of thyroid nodules, including those arising in HT. Two PTL cases in HT elderly patients are here described and discussed.

**Methods:**

FNC was performed in rapidly enlarged thyroid nodules of 2 elderly patients under ultrasound (US) control. FNC was used to prepare conventional cytologic smears, immunocytochemistry (ICC) and flow cytometry (FC) assessment of cell populations.

**Results:**

The above cases were diagnosed as well differentiated, small B-cell and diffuse large B-cell thyroid lymphomas, respectively, by means of FNC. The histological diagnoses were mucosa-associated non Hodgkin lymphoma (MALT) and diffuse large B-cell lymphoma (DLBCL), confirming FNC diagnoses, and patients were treated accordingly.

**Conclusions:**

FNC diagnosis of PTL is reliable and accurate; it may be conveniently used in the clinical practice since it provides indications for appropriate therapeutic procedures or diagnostic surgery, and avoids to treat benign nodules.

## Introduction

Primary thyroid lymphomas (PTLs) are a rare group of diseases that accounts for 5% of all thyroid malignant tumors, and approximately for 1/2% of all extra nodal lymphomas. Most PTLs are B- and T-cell non-Hodgkin lymphomas (NHLs), whereas primary thyroid Hodgkin lymphoma (HL) has been occasionally reported [1]. An high percentage of PTLs affects patients suffering from long standing Hashimoto Thyroiditis (HT), therefore PTL pathogenesis is probably related to chronic inflammation stimulation [2]. Although HT mainly affects women and adult patients, men and younger patients may also be affected [2-4]. PTL clinical signs include a rapidly enlarging mass in the thyroid area, variable hoarseness and dyspnea. NHL symptoms, such as weight loss, fever and nocturnal sweats, may also be present, although less frequently. Most PTLs are B-cell NHL, being mucosa-associated-lymphoid tissue (MALT) lymphoma and diffuse large B-cell lymphoma (DLBCL) the most frequently reported histotypes [5]. Treatment and prognosis depend on the specific subtype and staging; surgical treatment of localized tumors, radio and chemotherapy for low-grade and high-grade histotypes respectively is generally utilized [6]. A 5-year survival of 90% has been reported in correctly diagnosed and treated PTL patients [7]; therefore a timely and accurate diagnosis of PTL is mandatory for treatment and prognosis. Palpable neck masses are not a rare occurrence, some time representing a challenging diagnostic dilemma with unusual extrathyroidal masses [8,9]. Serological or cellular biomarkers would be of great diagnostic utility to distinguish benign from malignant thyroid nodules [10]. For instance, an increase in circulating levels of pro-angiogenic cytokines, as well as of bone marrow-derived endothelial progenitor cells (EPCs), has been observed in tumor patients [11-14]. Unfortunately, calcitonin is the only available biomarker to this purpose, and its utility is limited to the diagnosis of medullary thyroid carcinoma. Both molecular and functional studies have revealed that neoplastic cells remodel their Ca^2+ ^signaling machinery [11,15-18], thereby leading the notion that up-regulated plasmalemmal Ca^2+^-permeable channels might serve as alternative diagnostic markers of neoplastic transformation [18]. Unfortunately, these studies are yet to be performed in PTL. Fine-needle cytology (FNC) is the primary diagnostic tool [19-24] for all other nodular thyroid diseases. Inconclusive results are frequent and the application of molecular techniques to FNC has dramatically increased its sensitivity [24-32], including in HT cases with diffuse or nodular enlargement [31]. These advantages are enhanced in case of HT, which does not require surgical treatment, and even more in elderly patients, for whom surgery is generally more burdensome, complex and expensive than younger patients [33]. The aim of this study is to present 2 cases of PTL in elderly patients in which FNC pre-surgical diagnosis has contributed to a correct and differentiated treatment.

## Materials and methods

Between January 2010 and December 2012, 1.256 patients with thyroid nodules or diffuse swelling underwent FNC in the outpatient clinics of the Azienda Ospedaliera Universitaria, University of Salerno. Two of these patients were diagnosed with PTL; the first patient was a 66-year-old man who suffered from long standing HT. The gland had progressively enlarged in the last months causing dyspnea and difficulty in swallowing. The second was a 68-year-old woman with an undefined history of long standing goiter, who complained dyspnea, voice change and choking. Both patients underwent ultrasound (US)-guided FNC with rapid on-site evaluation (ROSE), as previously described [34-36]. The diagnostic procedure and its related risks were first discussed with the patients, who were also informed that 1 or 2 supplementary passes might have been needed, and an informed consent was obtained. Additional passes were used to prepare additional smears for immunocytochemistry (ICC) and cell suspensions in buffer solution to perform flow cytometry (FC) assessment of the cell populations. Monoclonal antibodies used for ICC were directed against thyroglobulin (TG), leucocyte common antigen (LCA), cytokeratin (CK), CD20, CD3, CD4, CD8, CD5, CD10, CD19, kappa and lambda light chains. Fluoresceinated antibodies for FC were: basic combinations of phycoerythrin (PE), perdin chlorophyll protein (PERCP) and fluorescein isothiocyanate (FITC) antibodies. Antibodies were purchased from Becton Dickinson (San José, CA). Antibodies clones, dilutions and technical details for both procedures were previously described [37,38]. In particular, FC data were interpreted accordingly in this specific clinical and anatomical setting [32,39]; with regard to the light chain evaluation for clonal assessment of the lymphoid cell populations, a percentage of the gated cells showing κ/λ unbalance ≤ 20% of the gated cells was considered evidence of clonality [32]. FC and ICC assessments were used to perform the final diagnoses and were included in the cytological reports.

## Results

**Figure 1:**
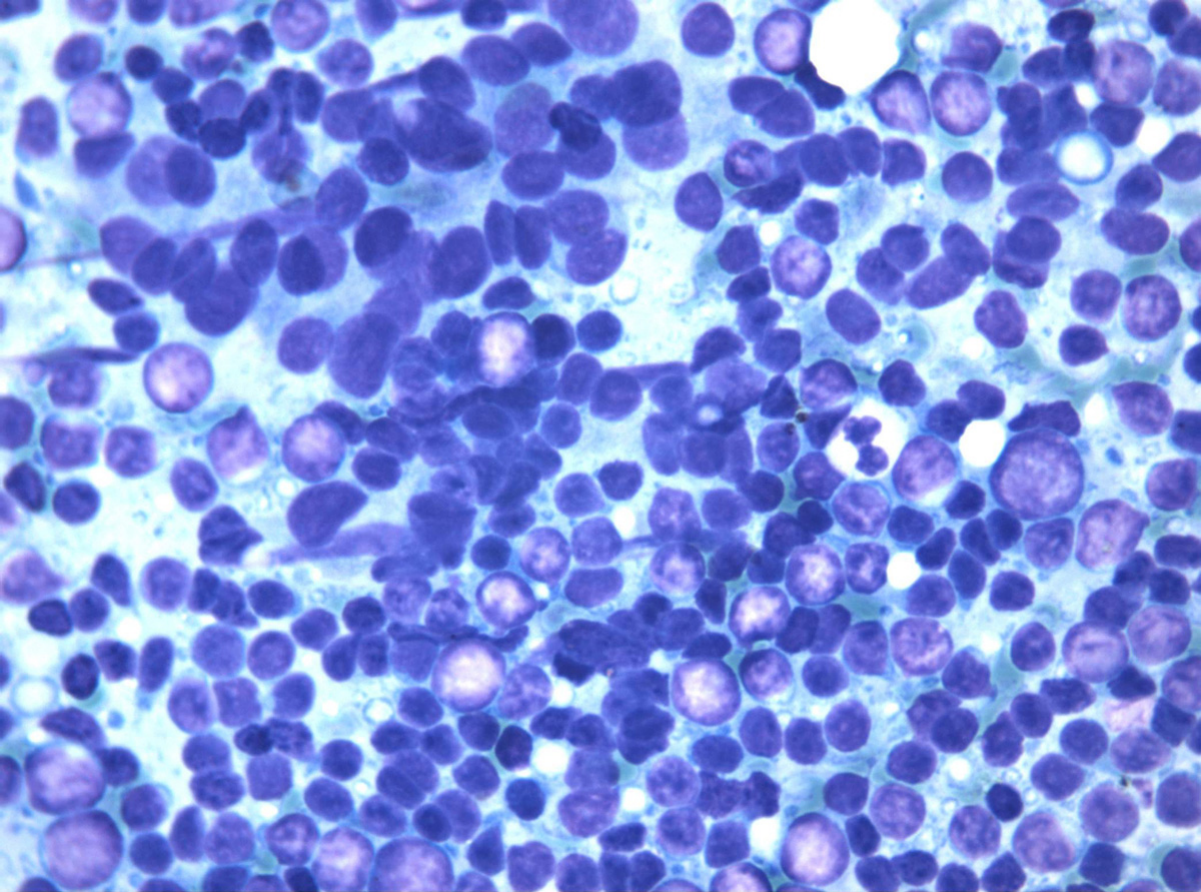
**Case 1 FNC smear showing monomorphic population of atypical lymphoid cells**. Thyroid follicular cells are absent. (Diff Quik stain 430X).

**Figure 2:**
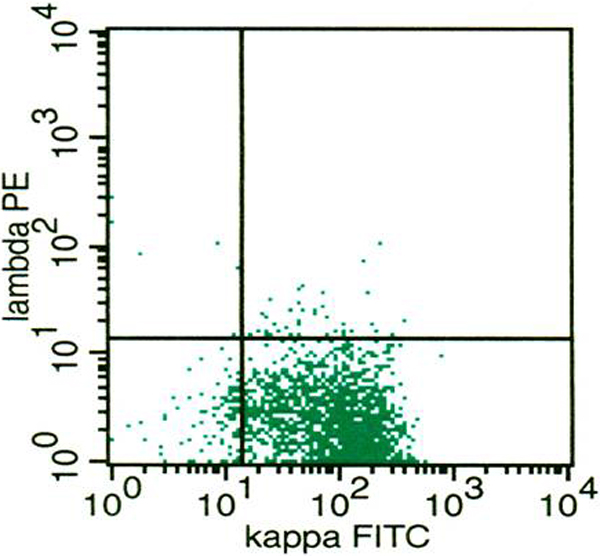
**Flow cytometry diagram showing Ä¸ light chain restriction**.

Case 1: At the time of FNC, US evaluation showed diffuse homogeneous enlargement of the right thyroid lobe, that measured 60 mm in diameter. The edges were smooth and the gland showed a homogeneous isoechoic pattern; FNC showed a dispersed population of immature medium size (Figure 1) lymphoid cells and small mature lymphocytes intermingled among the main cell population. Immature large lymphoid cells were roundish, 2 times the size of the mature lymphocyte and showed granular chromatin, nuclear membrane irregularities and 1 or 2 evident nucleoli. No thyroid follicular cells were observed. ICC showed LCA- and CD20-positivity in the main large cell population, CD3-positivity in small lymphocytes, and negativity for thyroglobulin. FC assessment of gated lymphocytes showed a large proportion of B-lymphocytes, identified by CD19. T-lymphocytes, identified by CD5, CD4 and CD8, were also present. B-lymphocytes were CD10-negative and showed k light chain restriction in 40% of the cell population (Figure 2). The differential diagnosis between PTL and HT with severe lymphoid infiltrate was taken into account. Nonetheless, HT was excluded because it generally shows lymphoid cells of variable size, plasma cells and tingible body macrophages, which were absent in this case. Moreover, the above reported k-light chain restriction was considered diagnostic for a B-cell type PTL arisen in HT. The patient received combination chemotherapy (CHOP) with local radiotherapy without surgical treatment; he is alive and free from signs of disease, to date.

**Figure 3:**
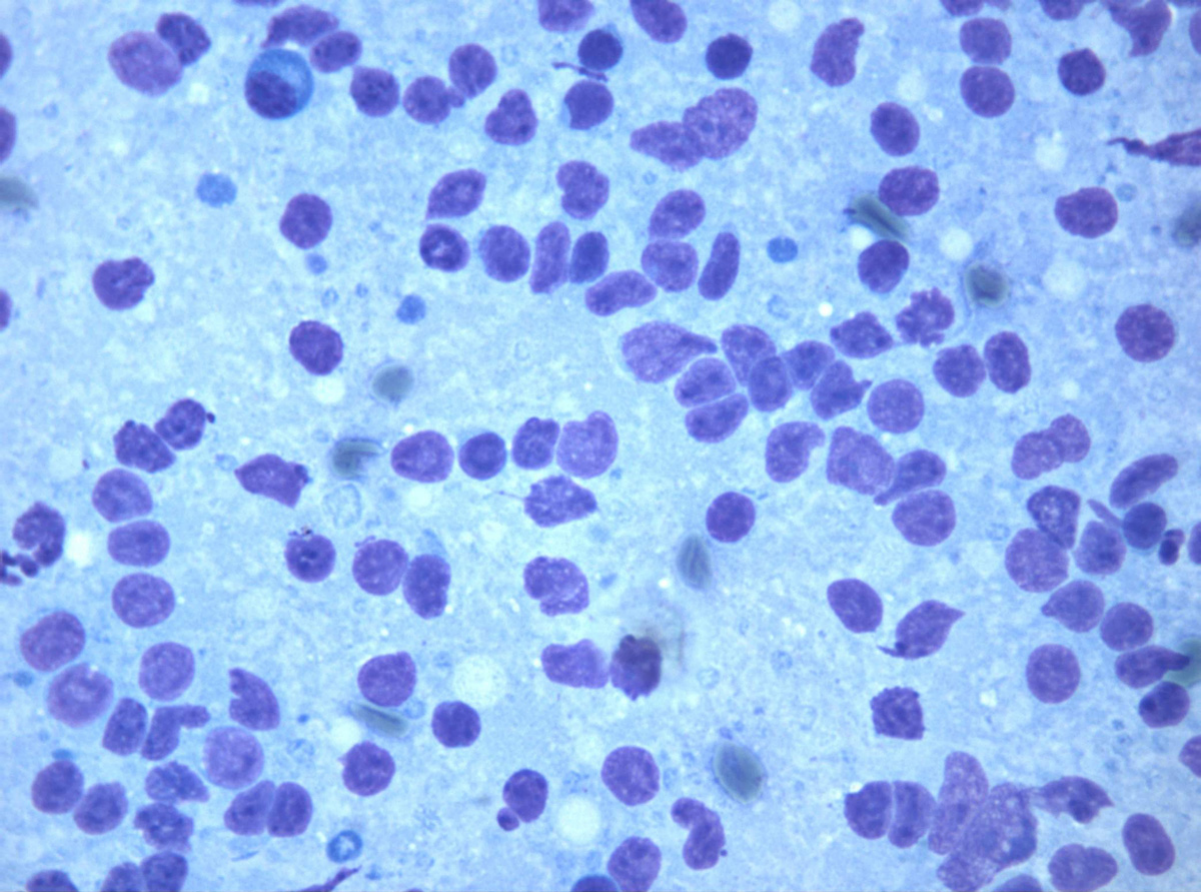
**Case 2 FNC smear showing atypical large lymphoid cells in a necrotic background (Diff Quik stain 430X)**.

**Figure 4:**
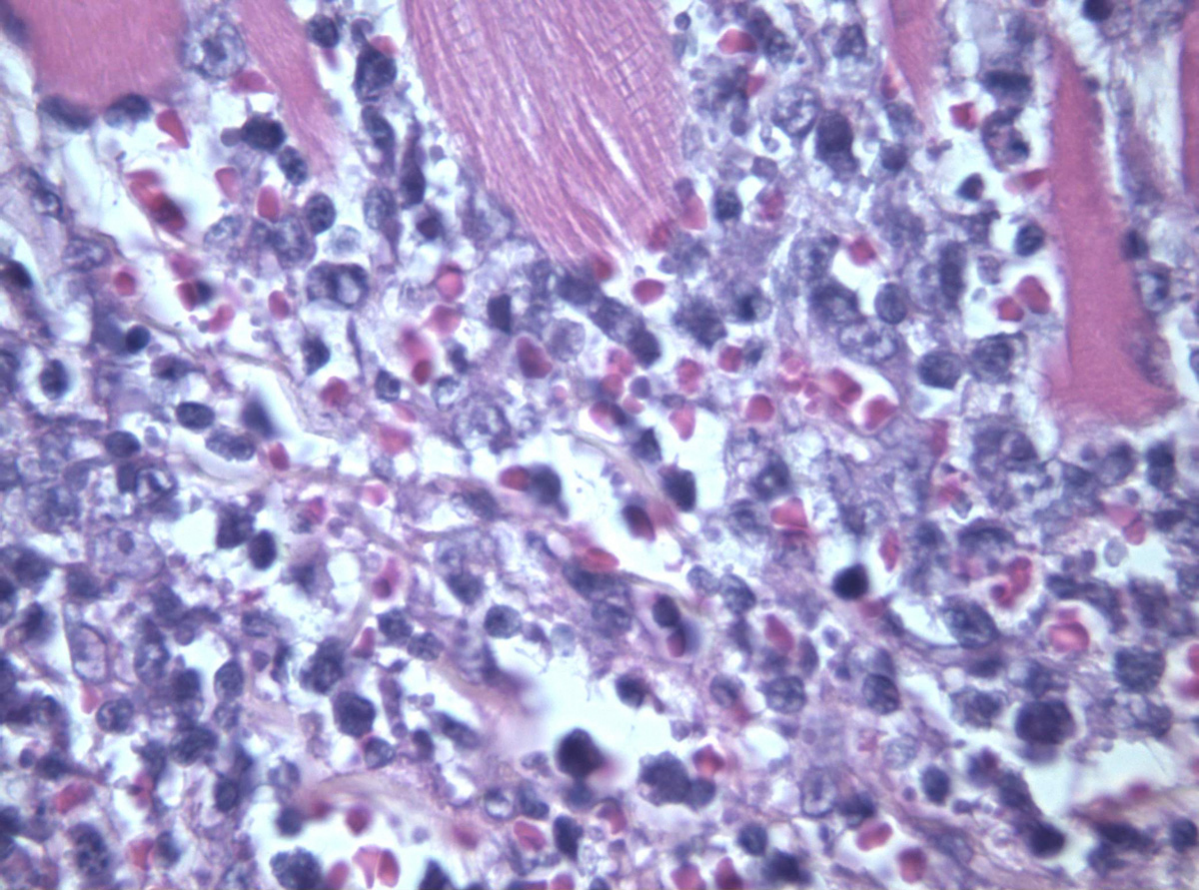
**Histological section of case 2 showing atypical lymphoid cells infiltrating the muscle (Hematoxylin-Eosin 430X)**.

Case 2: US showed an ill-defined, hypoechoic mass that, starting from the left thyroid lobe, infiltrated the surrounding neck structures including the sternocleidomastoid muscle. FNC showed an immature and dispersed large cell population. Cells showed evident nuclear abnormalities, coarse chromatin and 1 or 2 evident nucleoli (Figure 3). Scattered mature lymphocytes were present in the background, and no thyroid follicular cells were observed. ICC showed positivity for LCA, CD20 and for CD3 in small lymphocytes, being the main large cell population, and negativity for thyroglobulin. FC assessment of gated lymphocytes showed the large cells to be B-lymphocytes, identified by CD19. T-lymphocytes, identified by CD5, CD4 and CD8, were also present. B-lymphocytes were CD10-negative; light chains were not expressed. Because of the clinical presentation and the cytological features, a differential diagnosis of a possible anaplastic carcinoma was pointed out. Nonetheless, this possibility was excluded as ICC and FC data assessed the lymphoid B-cell origin of the neoplastic cells and FC did not detect light chain restriction. Therefore a cytological diagnosis of large B-cell NHL was pointed out. Notwithstanding the US and clinical presentation, lobectomy was performed and the following histopathological analysis confirmed the cytological diagnosis of NHL, which was morphologically and immunohistochemically consistent with diffuse large B-cell non-Hodgkin lymphoma (Figure 4). The patient received combination chemotherapy (CHOP) with local radiotherapy; she is alive and residual disease was detected at the last follow-up.

## Discussion

Primary thyroid lymphoma (PTL) is a lymphomatous process which develops in the thyroid without involvement of primary lymphoid organs or distant metastases at diagnosis [40]. As reported above, PTL mostly arises in the setting of autoimmune thyroiditis and takes an average of 20 to 30 years to develop after the onset of lymphocytic thyroiditis [41]. PTL frequently presents in IE/IIE clinical stages, with some clinical and imaging problems. A short history of a rapidly enlarging neck mass, often associated with dyspnea, difficulty in swallowing, or voice change, is the hallmark presentation of thyroid lymphoma [42]. These signs, as well as imaging procedures, may overlap with those of other benign and malignant thyroid pathologies and require an accurate pre-surgical diagnosis. FNC is the most frequently used procedure for the initial pathological diagnosis of thyroid nodules [43]. Conventional examination of cytologic smears yields inconclusive results that can be refined by testing for specific genetic alterations [26,27,44,45]. FNC has the highest levels of sensitivity and specificity [43], but studies have shown inconsistent results in the diagnosis of PTL. A correct diagnosis of PTL with FNC was made in 70/80% of cases [46], whereas other studies report that FNC was suggestive but not diagnostic in only 50/60% of cases [47,48]. The 2 study cases were both correctly diagnosed and are an example of the 2 main occurring histotypes, the cytological diagnostic difficulties and the importance of ancillary techniques applied to FNC. As reported above, in the first case the main differential diagnosis of HT with severe lymphoid infiltrate was pointed out. Indeed, the US presentation of HT is quite variable, ranging from normal thyroid parenchyma imaging to diffuse homogeneus enlargement or atrophy, up to uninodular or multinodular presentation in +/-50% of cases [49,50]. The US appearance of the nodules is also extremely variable; nodules may be hypo or hyper echoic, can have calcifications (micro and scattered, macro or eggshell calcifications), and may also show a hypoechoic halo. The cytological presentation showed the prevalence of lymphoid cells, which gave smears a lymph node-like appearance, including the prevalence of immature follicular centre cells. Finally, the possible presence of small clones of B cells with light chain restriction has been reported in HT, adding further diagnostic difficulties. On the other side, the monotonous cytological smear of immature lymphoid cells, the lack of thyroid follicular cells and the presence of large chain restrictions in most FC assessments of T gated cells strongly supported the diagnosis of PTL. In the second case, other diagnostic difficulties were encountered; in fact, clinical presentation and cytological features clearly indicated a malignant tumor, but a differential diagnosis of possible anaplastic carcinoma was pointed out. However, in this case FC did not show light chain expression, as it can occur in high grade B-cell NHL. Nonetheless, ICC and FC clearly indicated the lymphoid B-cell origin of the neoplastic cell. Therefore properly used ancillary techniques, together with clinical and imaging data and cytological features, allowed the diagnosis of PTL. This possibility enhances the cytological diagnosis not only in PTL cases, but also in the benign hyperplastic and nodular presentation of HT, in which surgery should not be considered neither for diagnostic or therapeutic purposes. PTL treatment is similar to other nodal NHLs. In case of intermediate or high-grade lymphoma, the best results are obtained with cyclophosphamide, doxorubicin, vincristine and prednisolone (CHOP) based chemotherapy. Radiotherapy is generally used after 3-6 CHOP courses in the form of modified mantle irradiation, including thyroid, bilateral neck, supraclavicular area and mediastinum [4]. Unfavorable prognostic factors include age (> 60 years), elevated levels of serum lactate dehydrogenase (LDH) and β_2 _microglobulin, extranodal sites involvement and III-IV stage [46]. Our patients received CHOP-based chemotherapy and radiotherapy; both are alive with (case 2) and without (case 1) signs of disease. In conclusion, the cytological diagnosis of PTL is reliable and accurate; it may be conveniently used in the clinical setting since it provides indications for diagnostic surgery, when needed, and avoids to treat HT cases.

## Competing interests

The authors declare that they have no competing interests.

## Authors' contributions

PZ, MV: conception and design, interpretation of data, given final approval of the version to be published; AC, EV, VDS, MV, AG, VDC: acquisition of data, drafting the manuscript, given final approval of the version to be published; PZ, MV: critical revision, given final approval of the version to be published.

## Declarations

Funding for this supplement came from personal funds.

This article has been published as part of *BMC Surgery *Volume 13 Supplement 2, 2013: Proceedings from the 26th National Congress of the Italian Society of Geriatric Surgery. The full contents of the supplement are available online at http://www.biomedcentral.com/bmcsurg/supplements/13/S2
